# Linking sensory biology and fisheries bycatch reduction in elasmobranch fishes: a review with new directions for research

**DOI:** 10.1093/conphys/cot002

**Published:** 2013-04-08

**Authors:** Laura K. Jordan, John W. Mandelman, D. Michelle McComb, Sonja V. Fordham, John K. Carlson, Timothy B. Werner

**Affiliations:** 1 Ecology and Evolutionary Biology, University of California, Los Angeles, Los Angeles, CA 90095, USA; 2 John H. Prescott Marine Laboratory, New England Aquarium, Boston, MA 02110, USA; 3 Marine Science, Ocean Classrooms, Boulder, CO 80301, USA; 4 Shark Advocates International, a project of The Ocean Foundation, Washington, DC 20036, USA; 5 Southeast Fisheries Science Center, NOAA Fisheries Service, Panama City, FL 32408, USA; 6 Consortium for Wildlife Bycatch Reduction, New England Aquarium, Boston, MA 02110, USA

**Keywords:** Bycatch reduction, elasmobranch fishes, fisheries, sensory

## Abstract

Incidental capture, or bycatch, in fisheries represents a substantial threat to the sustainability of elasmobranch populations worldwide. Consequently, researchers are increasingly investigating elasmobranch bycatch reduction methods, including some focused on these species' sensory capabilities, particularly their electrosensory systems. To guide this research, we review current knowledge of elasmobranch sensory biology and feeding ecology with respect to fishing gear interactions and include examples of bycatch reduction methods used for elasmobranchs as well as other taxonomic groups. We discuss potential elasmobranch bycatch reduction strategies for various fishing gear types based on the morphological, physiological, and behavioural characteristics of species within this diverse group. In select examples, we indicate how an understanding of the physiology and sensory biology of vulnerable, bycatch-prone, non-target elasmobranch species can help in the identification of promising options for bycatch reduction. We encourage collaboration among researchers studying bycatch reduction across taxa to provide better understanding of the broad effects of bycatch reduction methods.

## Abstract (precis)

Incidental capture, or bycatch, of elasmobranchs (sharks, skates, and rays) threatens populations worldwide. In this review, elasmobranch sensory biology and ecology are explored to identify potential species- and fishery-specific bycatch reduction techniques for a variety of fishing gear types.

## Introduction and background

As populations of marine species face increasing declines due to anthropogenic activities, concern over wasteful loss of biomass as incidental catch, or ‘bycatch’, is growing on a global scale (Hall *et al.*, 2000; Lewison *et al.*, 2004; Kelleher, 2005). Levels and composition of bycatch, which can be defined as retained or discarded non-target species as well as unobserved mortality prior to or following landing/release (Gilman, 2011), vary spatially, temporally, and with gear type. With the current level of under-reporting, lack of species identification, unobserved mortality, and incomplete understanding of at-vessel and post-release mortality, the total global bycatch mortality (all species) is difficult to determine, but estimates range from 7.0 to 38.5 million tonnes annually, accounting for up to 40.4% of total catch (Alverson *et al.*, 1994; Kelleher, 2005; Davies *et al.*, 2009). A significant number of marine species affected by high bycatch rates are classified by the International Union for Conservation of Nature (IUCN) as threatened (vulnerable, endangered, or critically endangered).

Research aimed at reducing bycatch of marine mammals, sea birds, and sea turtles has grown since the late 1980s, while prioritization for elasmobranch (shark, skate, and ray) bycatch reduction has lagged behind (Werner *et al.*, 2006). Recent reports of fishery-driven global declines in many elasmobranch populations (e.g. Dulvy *et al.*, 2008; Camhi *et al.*, 2009), high rates of incidental capture in certain fisheries (e.g. Mandelman *et al.*, 2008), high at-vessel mortality rates observed in some species (e.g. Morgan and Burgess, 2007), and K-selected life history traits (Hoenig and Gruber, 1990; Cortés, 1999, 2002; Heppell *et al.*, 1999) have elevated the priority of elasmobranch bycatch reduction within fisheries management. Elasmobranchs differ from other focal species, such as sea birds or dolphins, in that directed elasmobranch fisheries are widespread and sale of incidental elasmobranch catch is profitable in many regions where markets for fins, meat, and other products are accessible (Davies *et al.*, 2009). Nevertheless, incidental capture is detrimental to populations of elasmobranch species, many of which fulfil important ecological roles in their communities (Stevens *et al.*, 2000). Moreover, elasmobranch bycatch and depredation can compromise crew safety and decrease profitability through reduced target catch and gear damage (Gilman *et al.*, 2008).

Elasmobranchs are caught in virtually all types of fishing gear, with rates dependent on the species, size class, gear selectivity, location, and extent of effort, among other factors. Fishing gears can be classified in a variety of ways, including those with nets (e.g. trawls, gill nets, and purse seines), and those with baited hooks. Although trawls and gill nets have variable mesh sizes to improve selectivity for target species, they can be highly indiscriminate for non-target species, capturing any organism that does not fit through the mesh or cannot escape entanglement, and are usually associated with high levels of bycatch (Alverson *et al.*, 1994). While hook fisheries depend on attraction of target predatory species to bait, they can also result in high levels of incidental catch (Gilman *et al.*, 2008). Pelagic longlines, gill nets, and trawls are responsible for the highest annual reported biomass of direct and indirect global elasmobranch catch (Bonfil, 1994; Gilman *et al.*, 2008; Mandelman *et al.*, 2008; Molina and Cooke, 2012).

Over the past 30 years, researchers have identified successful methods to reduce bycatch of various taxa through modifications to fishing practices and gear that often involve sensory-based strategies, including acoustic, visual, and chemical signals. Although typically aimed at one species or group, bycatch mitigation methods can influence bycatch rates of other taxa encountering the same fishing gear. For example, bottom trawls outfitted with excluder or bycatch reduction devices (BRDs), such as turtle excluder devices (TEDs) designed to release sea turtles, can also decrease elasmobranch bycatch (Brewer *et al.*, 1998, 2006; Fennessy and Isaksen, 2007). The opposite can also be true, where modifications that decrease bycatch of one species can increase catch of another. For example, a shift from dolphin-associated purse seine sets towards using fish aggregating devices (FADs) has decreased dolphin bycatch; however, bycatch of sea turtles, sharks, and non-target teleost fish increases with use of FADs (Lewison *et al.*, 2004; Gilman, 2011). For these reasons, it is important to understand the effects of modifications to fishing practices and gear on all target and non-target species.

Several fishing practices and gear modifications have been identified to reduce elasmobranch bycatch in a variety of fisheries, although most direct research on this topic has been applied to hook gear (Molina and Cooke, 2012). For example, the use of circle rather than J hooks, fish instead of squid bait, monofilament in place of steel leaders, and setting at deeper depths for shorter lengths of time can contribute to decreased catch and/or mortality of elasmobranchs on pelagic longlines (Watson *et al.*, 2005; Ward *et al.*, 2009; Gilman, 2011). However, the high level of variability among studies highlights the need for shark-specific controlled experiments to provide more definitive results (Godin *et al.*, 2012). For net fisheries, limits on mesh size, increased tension (e.g. of gill nets), addition of excluder devices (in trawls), and modifications to FAD construction and use (for purse seining) can also reduce elasmobranch bycatch (Brewer *et al.*, 2006; Thorpe and Frierson, 2009; Schaefer and Fuller, 2011). The emerging field of sensory-based deterrents directed at elasmobranchs has centred on the electrosensory system (see O'Connell *et al.*, 2012). While the use of electric and magnetic deterrents to repel elasmobranchs warrants further research and development, it is important to explore additional sensory pathways for bycatch reduction potential.

Bycatch mitigation research has generally aimed either to reduce contact with gear or to facilitate escape once contact occurs (Werner *et al.*, 2006). For elasmobranchs, immediate (at-vessel or capture) and post-release (delayed or discard) mortality rates vary widely both within and between species (Morgan and Burgess, 2007; Skomal and Bernal, 2010). Capture mortality ranges from 15 to 100% by species and gear type, among other factors (e.g. Beerkircher *et al.*, 2002; Moyes *et al.*, 2006; Mandelman and Farrington, 2007; Campana *et al.*, 2009; Thorpe and Frierson, 2009; Morgan and Carlson, 2010). Although estimated short-term post-release survivorship appears high in some species (Moyes *et al.*, 2006; Campana *et al.*, 2009), unaccounted sublethal effects (e.g. cryptic injury, latent pathology, and impaired feeding) can ultimately prove fatal for individuals, with possible population-level consequences (e.g. Borucinska *et al.*, 2002; Frick *et al.*, 2010; Skomal and Mandelman, 2012). For further discussion of this important topic, we refer the reader to Skomal and Bernal (2010). For the purposes of this review, emphasis is placed on reducing interactions of elasmobranchs with gear to decrease both immediate and delayed mortality that may result from contact with fishing gear targeting other species.

Elasmobranchs occupy a wide variety of ecological niches and possess diverse sensory and behavioural adaptations. Gaining a better understanding of this diversity may hold potential for refining or redirecting previously tested methods or developing new, more effective bycatch reduction strategies. Our objectives are as follows: (i) to summarize current knowledge of elasmobranch sensory biology and feeding behaviour related to potential interactions with fishing gear; (ii) to review bycatch reduction research that is directly or indirectly related to elasmobranchs; and (iii) to explore new research directions that may be useful in reducing bycatch in example species associated with some or all of the following factors: low or rapidly declining populations; high incidental capture rates in a given fishery or fisheries; and high or unknown mortality due to capture and handling. Our ultimate goal is to suggest promising new directions for research to reduce unintended interactions between elasmobranchs and fishing gear.

## Sensory biology

**Figure 1.**
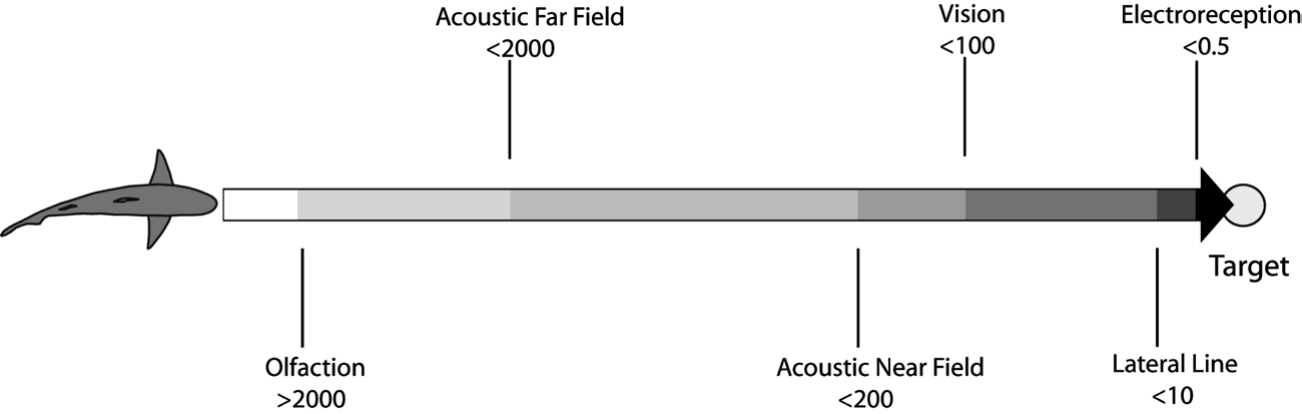
Relative transmission/detection distances (in metres) associated with potential sensory signals emitted by a target, such as a prey item. As the elasmobranch approaches the target, additional types of sensory information become available for detection using different sensory modalities. All distances approximate a best-case scenario; however, actual distances are influenced by environmental variables and interspecific variation in sensory systems.

Elasmobranchs use a variety of information from their environment to navigate and locate other organisms (prey, predators, and conspecifics). Chemical, mechanical, visual, and electrical signals can be detected by peripheral sensory systems, interpreted by the central nervous system, and result in physiological and behavioural responses. Use of these signals to locate food may vary by species, distance, prey type and environmental factors. Chemical and acoustic signals propagate long distances from the source, followed by visual, water flow, and finally electrical information at short range (Fig. 1). We review current knowledge of elasmobranch sensory biology to highlight areas where sensory signals may be useful for bycatch reduction. Comparisons with teleosts are included where possible to identify differences that may enhance gear selectivity for target species. We also include examples of new and existing bycatch reduction methods aimed at each sensory system for elasmobranchs as well as other taxa. Our priority is to identify modifications to fishing gear that will reduce bycatch with little or no effect on target catches. For a more thorough introduction to elasmobranch sensory systems, see Gardiner *et al.* (2012) and Collin (2012).

### Chemical detection

Elasmobranchs are sensitive to chemical signals via taste, common chemical sense, and olfaction. In seawater, chemical signals as odours can be carried great distances from their source, forming complex plumes, eddies, and intermittent signals (e.g. Webster, 2007). The distribution of odour signals depends on water flow and can be influenced by currents, topography, and the movements of organisms. Olfaction is important, if not essential, for initiation of search behaviour in both elasmobranchs and teleosts; however, additional sensory input is typically needed for location of a potential food source (Atema *et al.*, 1980; Gardiner and Atema, 2007). For example, tracking of odours is accomplished with both the olfactory sytem and the lateral line system (see ‘Mechanical detection: water flow’ below) in tandem (Gardiner and Atema, 2007). Despite the enlarged olfactory organs of elasmobranchs, sensitivity to specific amino acids is similar in threshold (10^−9^ mol/l) and amino-acid type in both elasmobranchs and teleosts (Meredith and Kajiura, 2010). Differences in behavioural thresholds and sensory organ thresholds are probable, so it is essential that behavioural experiments provide information on sensitivity as well as the nature of the response (attraction/repulsion). Olfaction-related behavioural experiments have been limited by the difficulty of obtaining precise concentration data at the receptor of free-swimming animals (Gardiner *et al.*, 2012).

Sharks display attraction to odours derived from fish and invertebrates (potential prey), particularly those from stressed fish, and repulsion (although variable) to human sweat (Tester, 1963). Some sharks also display an adverse reaction to chemicals derived from a potential predator (Rasmussen and Schmidt, 1992) and toxins such as those produced by the moses sole, *Pardachirus marmoratus* (Clarke, 1983). Hundreds of chemical compounds have been studied as shark deterrents (e.g. Gilbert, 1968), yet researchers continue efforts to identify one that is effective across a variety of species. Both synthetic surfactants and semiochemicals produced by sharks appear promising (Smith LJ Jr, 1991; Sisneros and Nelson, 2001; E. Stroud, personal communication). Chemical deterrents are an intriguing option to reduce elasmobranch interactions with fishing gear because of their importance in early detection and arousal for prey search behaviours, although control over the chemical dispersion rates remains a challenge. Future studies should be aimed at identifying a chemical that masks attractive odours or elicits a repulsion response in elasmobranchs without altering behaviour of targeted species. Behavioural reactions to conflicting attractive and repulsive odours may be unpredictable, so future research should also include the use of chemical deterrents with artificial bait. Alternatively, attracting elasmobranchs to sites away from fishing gear via ‘remote attracting devices’ is another promising area of current research (Dagorn, 2010). In this scenario, the attractant odour should be specific to elasmobranchs, so that targeted fish are not also attracted. Behavioural and field experiments to test the responses of both target and non-target species are essential in determining effectiveness of candidate deterrents or attractants.

Research on chemical signals for bycatch reduction in other species groups has met varying levels of success. Shark liver oil slicks dripped at the surface during longline setting reduced seabird interactions with fishing gear without influencing fish catch (Pierre and Norden, 2006). However, no noxious chemicals have been identified for successfully reducing sea turtle or marine mammal bycatch (Gearin *et al.*, 1988; Southwood *et al.*, 2008). Experiments with bait type have revealed that using fish instead of squid bait reduces both sea turtle and shark bycatch, without decreasing target catch (e.g. Watson *et al.*, 2005; Gilman, 2011). With sea turtles, the difference in catch is attributed to bait-dependent differences in capture strategy/bait consumption (Gilman, 2011); however, it is not known whether chemical signals emitted by the bait or consumption behaviour contribute to observed decreases in catch rate of elasmobranchs.

### Mechanical detection: sound

Mechanical stimuli can be detected by the hearing apparatus, mechanosensory system (discussed separately under ‘Mechanical detection: water flow’ below), and skin (cutaneous receptors) of elasmobranchs. Sounds travel quickly in water and can propagate from metres to kilometres in the ocean, depending on the frequency of the signal. Sound signals consist of pressure and particle displacement components that fall off at different rates with distance from the source, defining the acoustic near and far fields, and may be complicated by interactions with surface or ground interfaces and structures (Myrberg, 2001). Elasmobranchs are sensitive to the particle displacement component of sounds within the range of 20–1000 Hz (Casper and Mann, 2006, 2010), and sharks have been attracted from beyond visible distances by low-frequency, irregularly pulsed sounds, particularly those ranging within 25–50 Hz (Nelson and Gruber, 1963; Banner, 1967; Myrberg *et al.*, 1972, 1976), although laboratory studies have raised questions over sharks' capability of detecting sounds in the acoustic far field (see Casper and Mann, 2006). Abrupt and high-intensity sounds (10× ambient noise) elicited fright or withdrawal responses in sharks within 10 m of the source; however, habituation was observed (Myrberg, 2001). Teleost fishes, such as tunas, targeted by pelagic longlines are also sensitive to low-frequency sounds below 1000 Hz, particularly within 200–500 Hz (Iversen, 1967, 1969; Moein Bartol and Ketten, 2006). Some teleosts are equipped with bony connections to the swim bladder that provide them with a mechanism for detection of the pressure component of sound (See Popper, 2003).

Acoustic cues in the form of high-frequency acoustic pingers can reduce bycatch of some marine mammals and seabirds in gill nets; however, concerns remain over habituation and association of food with pingers, and potential hearing damage associated with higher decibel acoustic harassment devices (e.g. Melvin *et al.*, 1999; Bordino *et al.*, 2002; Barlow and Cameron, 2003). No influences of pingers on catch rate of elasmobranchs have been reported, and they are not expected, given that the frequencies used are beyond the range of known elasmobranch hearing. Predator sounds have also been tested on the behaviour of cetaceans, yielding mixed results (Fish and Vania, 1971; Scordino and Pfeifer, 1993). Seismic air guns were found to keep sea turtles away from a power plant intake, but the intensity of sound required was likely to cause damage to marine organisms at close range (Popper, 2003). Current research seeks to use frequencies that may be more benign. Growing concerns over increasingly high levels of sound in the ocean are a necessary consideration for development of acoustic deterrents (Southwood *et al.*, 2008). The increasing levels of anthropogenic background noise and decreasing absorption of low-frequency sounds with ocean acidification (Hester *et al.*, 2008) are likely to be major factors influencing organisms' abilities to sense and respond to biologically meaningful sounds and raise concern over the introduction of additional sounds into the ocean for bycatch reduction purposes.

### Visual detection

The elasmobranch visual system has been comparatively well studied, and its structure and function rival that of higher vertebrates (e.g. Hart *et al.*, 2006; Lisney *et al.*, 2012). Vision is a dominant sensory domain and, in clear waters, can extend tens of metres, providing continuous, near-instantaneous information relative to orientation, movement, habitat, predators, prey, and conspecifics.

The intensity of ambient light in the aquatic environment is dynamic and can vary nine orders of magnitude based on time of day, angle of incidence, scatter, and seasonality (Lythgoe, 1979; McFarland, 1990). The maximal transmission of light occurs at short wavelengths in deep-sea and clear, open-ocean environments (blue), at intermediate wavelengths in coastal waters (green), and at longer wavelengths in estuarine and freshwater (yellow–red; Jerlov, 1968). Positioned within the retina of most elasmobranchs are both rods, which confer sensitivity and resolution in low-light conditions, and cones, which confer sensitivity in bright-light conditions, higher acuity, and potential colour discrimination. The proportions of rods and cones vary by species according to ecological factors, with deeper dwelling species possessing fewer to no cones (e.g. Bozzano, 2004; Hart *et al.*, 2006). The spectral tuning of rod visual pigments correlates with depth; deeper dwelling species have more blue-shifted sensitivities, in contrast to those living nearer to the surface. Cone spectral sensitivity generally matches ambient environmental spectra, yet sharks studied to date appear to be monochromats and may therefore be colourblind (Gruber, 1975; McComb *et al.*, 2010; Hart *et al.*, 2011), although potentially unexplored mechanisms involving comparisons of rod and one cone input may allow sharks to achieve coloir discrimination, as is suggested in pinnipeds (Griebel and Peichl, 2003). In contrast to sharks, some batoids (skates, rays, and guitarfish) possess multiple spectrally distinct cone pigments, suggesting the capacity for colour vision (Hart *et al.*, 2004; Theiss *et al.*, 2007). In terms of behavioural responses, various species of sharks have displayed attraction to certain colours and high-contrast objects (McFadden and Johnson, 1971), although colour discrimination has only recently been conclusively demonstrated in one ray species (Van-Eyk *et al.*, 2011).

The elasmobranch eye possesses distinct morphological and physiological adaptations that maximize visual function in differing habitats. Adaptations include variation in eye size, eye positioning, mobile pupils, elaborate pupillary operculae, and reflective retinal media (Walls, 1942). Elasmobranchs generally possess relatively large visual fields, up to 360 degrees, with varying amounts of binocular vision facilitating depth perception (McComb and Kajiura, 2008; McComb *et al.*, 2009). Species that inhabit the upper 200 m of the open ocean typically have larger eyes and associated areas of the brain, and faster temporal resolution, and are thought to rely more heavily on vision for locating prey (Kajiura *et al.*, 2010; McComb *et al.*, 2010).

Differences between the vision of elasmobranchs (and other bycatch species) and that of target teleosts may prove useful for bycatch mitigation. Several gear modifications to alter the visual appearance of gear have been investigated, particularly in the pelagic environment. Light sticks are frequently used to attract target fish to bait on pelagic longlines, and modifying them is a potential mechanism to reduce sea turtle bycatch (Crognale *et al.*, 2008; Southwood *et al.*, 2008). The cone sensitivities of shark species range from 480 to 561 nm (Hart *et al.*, 2004, 2011; McComb *et al.*, 2010), while striped marlin (*Tetrapturus audax*) are reported to have multiple visual pigments with peak sensitivity at 436, 488, and 531 nm (Fritches *et al.*, 2003), and adult yellow fin tuna (*Thunnus albacares*) have peaks at 426 and 483 nm (Loew *et al.*, 2002). The use of chemical lights outside the range of known shark sensitivity and within range of target fishes may help reduce elasmobranch attraction to gear.

Electroluminescent light sticks or light-emitting diodes (LEDs) offer the opportunity to set specific spectral characteristics as well as flicker or flash rates. Temporal resolution, measured as the critical flicker fusion frequency (CFF) or flicker fusion frequency (FFF), varies among species and presents a novel opportunity to explore manipulation of light flicker on longlines in efforts to reduce attraction by non-target species. Flicker and moving stimuli are detected readily by the visual system and, therefore, knowledge of the CFF of target and non-target species would be beneficial for designing bycatch reduction devices. The CFF for a given species varies with temperature and with light/dark adaptation of the eye, thus future experiments should examine the CFF of species in field conditions (e.g. dark or scotopic adaptation and relevant temperatures). The night-time FFF ranges from 30 to 60 Hz in teleost fishes (Horodysky *et al.*, 2008, 2010), including swordfish (Fritches *et al.*, 2005) and tuna (Bullock *et al.*, 1991; Brill *et al.*, 2005), while the scotopic CFF in elasmobranchs ranges from 16 to 25 Hz (McComb *et al.*, 2010). Flicker rates higher than 30 Hz could increase attraction by teleosts, while simultaneously decreasing attraction to species with lower CFF, such as sharks and leatherback sea turtles (Crognale *et al.*, 2008), to which the light would appear steady and dimmer. The determination of CFF at various wavelengths in relevant laboratory conditions for both target and non-target species, coupled with field-testing, would improve our understanding of the applicability of this technology.

Altering the appearance of the bait itself by dying it blue has been found to decrease seabird, though not sea turtle, bycatch in pelagic longline fisheries (e.g. Brothers *et al.*, 1999; Swimmer *et al.*, 2005). The effects of dyed bait on shark catch were not specifically reported in these studies and remain unknown. Lydon and Starr (2005) reported use of additional colours of dye, including green and red, in commercial longline fisheries to increase target catch, though effects of these practices on shark catch are also unknown. Bird-scaring or Tori lines provide an effective visual signal to keep birds from longlines during deployment when they are vulnerable to becoming hooked and drowned by the gear (Alexander *et al.*, 1997). The use of monofilament leaders, which are less visible to teleosts than steel, can increase target species catch while simultaneously decreasing shark catch (Ward *et al.*, 2008), although concurrent gear adjustments are required to avoid increasing seabird bycatch (Gilman *et al.*, 2008). Even though the number of landed sharks decreases, use of monofilament is unlikely to decrease the number of sharks interacting with gear, resulting in more hooks being bitten off and potential delayed mortality (Afonso *et al.*, 2012).

For net-based fisheries, several modifications have been investigated, including increasing the visibility of the net, adding fake predator models, or increasing visibility of escape hatches. Illumination of gill nets can increase visibility of the net, thereby allowing some species, including sea turtles, to avoid entanglement (Wang *et al.*, 2010). Recent field trials suggest that LED lights in or near the ultraviolet range can reduce both sea turtle and elasmobranch bycatch in gill nets while increasing target species catch (J. Wang, personal communication). Visual predator models have also successfully decreased sea turtle bycatch in gill nets, although fish catch, including target species and elasmobranchs, was also reduced (Wang *et al.*, 2010). Difference in colour (white or black) of an excluder grate to reduce the number of spiny or piked dogfish, *Squalus acanthias*, captured by trawl did not influence shark behaviour, with both colours resulting in a dramatic decrease in spiny dogfish bycatch (Chosid *et al.*, 2012).

### Mechanical detection: water flow

The mechanosensory systems of elasmobranchs include the lateral line canals, superficial neuromasts, spiracular organs, and vesicles of Savi. Mechanoreceptor type and distribution vary by species (Maruska, 2001; Jordan, 2008). Components of these systems are sensitive to the velocity or acceleration of water flow around the body or within canals. Signals detected by the lateral line system of elasmobranchs are low frequency (<200 Hz), with velocities and accelerations respectively in the micrometre per second and millimetre per square second range (Maruska and Tricas, 2004). Hydrodynamic signals within this range are important in prey detection for orienting to currents (rheotaxis), in tracking odour plumes (Gardiner and Atema, 2007), and in locating weak water jets similar to those produced by prey (Montgomery and Skipworth, 1997; Jordan *et al.*, 2009). However, compared with teleosts, relatively little is know about the mechanosensory systems of elasmobranchs. In teleosts, water motion caused by currents, other organisms, and the individual itself can provide information used for complex behaviours, including navigation, hydrodynamic imaging, predator and prey localization, and schooling (Coombs and Montgomery, 1999). Water flow signals from a dipole source, such as a prey organism, are relatively short ranging, while currents can provide information essential for navigation and locating distant prey (see ‘Chemical detection’ above). Functional consequences of differences in anatomical structure between elasmobranchs and bony fishes, such as the distribution of neuromasts within lateral line canals and the presence of non-pored canals, as well as morphological variation between species of elasmobranchs, are not well understood.

Bycatch mitigation strategies using hydrodynamic signals have been largely unexplored. We introduce a potential application for bottom trawls, particularly those targeting invertebrates. Elasmobranchs, including both skates and sharks, have been observed to respond only upon contact with trawl gear (Queirolo *et al.*, 2012). Water jets directed downward and forward of the gear could elicit an earlier response, allowing elasmobranchs an opportunity to avoid capture in the trawl.

### Electrical detection

The ampullary electrosensory system of elasmobranchs consists of an array of pores at the surface connected to sensory cells by gel-filled canals and is highly sensitive to low-frequency electrical stimuli produced by both non-biological and biological sources. Electric fields within the range of detection by elasmobranch electroreceptors are created by temperature gradients (Brown, 2010), currents moving through the Earth's magnetic field (Kalmijn, 1971), geomagnetic anomalies (Klimley, 1993), underwater electrical cables (Koops, 2000), and bioelectric fields (Kalmijn, 1971). Electric field gradients fall off quickly in seawater, thus weak electric signals, such as those produced by live prey, are detectable only at short range. This distance is influenced by the signal strength and orientation, as well as temperature and salinity, which determine the resistivity of seawater (Kalmijn, 1971). Species with differing evolutionary histories, feeding ecologies, and electrosensory system morphologies have thus far shown similar sensitivity to electric field strengths, with behavioural responses observed below 1 nV/cm from a maximal distance of 30–40 cm (Kajiura and Holland, 2002; Kajiura, 2003; McGowan and Kajiura, 2009; Jordan *et al.*, 2011). While the minimal electric field strength eliciting an orientation response is similar across species, variation in the number and distribution of electrosensory pores, along with recent behavioural work, suggest that different species rely on the electrosensory system to varying degrees to locate and capture prey (Kajiura *et al.*, 2010; Gardiner, 2011). Coastal species and those feeding on benthic prey are most likely to rely heavily on the electrosensory system (Kajiura *et al.*, 2010).

The electrosensory system was lost during bony fish evolution, and is not present in species typically targeted by fishing activities. Thus, the production of electric signals detectable by elasmobranchs but not teleost fishes has been the focus of several recent deterrent-based bycatch reduction studies (e.g. Stoner and Kaimmer, 2008). Magnets, lanthanide metals/alloys, and powered electrical devices have been evaluated as potential deterrents to prevent consumption of baited hooks or to act as a barrier that could prevent capture in nets (e.g. Marcotte and Lowe, 2008; Robbins *et al.*, 2011). All three types of electrical deterrents produce electric signals different from those created by living things and may over-stimulate the sensitive electrosensory system, eliciting an aversion response.

Laboratory and field experiments with lanthanide metals and magnets have reported conflicting results in eliciting avoidance behaviour, decreasing shark catch, and observing habituation (e.g. Kaimmer and Stoner, 2008; Wang *et al.*, 2008; Rigg *et al.*, 2009; Tallack and Mandelman, 2009; O'Connell *et al.*, 2011; Robbins *et al.*, 2011; Hutchinson *et al.*, 2012). Variation in the response to lanthanide metal deterrents has been observed both between and within species (Jordan *et al.*, 2011). Factors such as hunger level and presence of potential competitors may contribute to variation in both laboratory and field settings (Brill *et al.*, 2009; Robbins *et al.*, 2011). In the presence of conspecifics, the dusky smoothhound, *Mustelus canis*, switched from avoiding food attached to a lanthanide metal (when alone) to preferring it (Jordan *et al.*, 2011). Inter-specific differences have been attributed to ecological variables, with greater deterrent success being found for coastal, benthic-feeding species or age classes (Rigg *et al.*, 2009; Hutchinson *et al.*, 2012). Future studies may achieve greater success when focusing on species that are likely to rely heavily on electroreception to locate prey and are rarely observed to feed in groups.

Deterrents aimed at the electrosensory system present various economic and logistical challenges (see O'Connell *et al.*, 2012). For example, lanthanide metals dissolve in seawater, requiring frequent replacement, which increases the time spent modifying gear and exacerbates cost concerns for materials. Magnets can present additional challenges for handling on board ships and during deployment/recovery of gear. Powered electronic devices have been marketed as shark-attack deterrents, although their effectiveness has rarely been evaluated experimentally (Huveneers *et al.*, 2012). One area of current research is focused on developing battery-powered electrical devices that can be affixed to hook-and-line fishing gear to reduce elasmobranch bycatch (S. Kajiura, personal communication). Whether or not these are more cost effective remains to be seen. High-powered electric fields to exclude sharks from larger areas require considerable amounts of energy and also achieve variable effectiveness (Smith ED, 1991; Marcotte and Lowe, 2008). High-powered electric fields have shown promise in reducing seal depredation on fish caught by gill net (Forrest *et al.*, 2009), indicating that further development of this technology could be useful for reducing gear interactions of multiple non-target taxa.

### Sensory modality convergence during feeding events

As an elasmobranch approaches its prey, more sensory cues become detectable (Fig. 1). In the ocean, sound and odour signals entrained in currents can potentially travel long distances from the source and may contain initial information directing marine predators toward food. Depending on water clarity and light levels, vision can aid in prey localization up to several tens of metres away. Electric and hydrodynamic signals in seawater have a short range, and typically elicit orientations by elasmobranchs within a metre of the source.

Multisensory integration is fundamental to the formation of unified sensory perception and is present in a wide range of taxa (e.g. Rowland and Stein, 2008). This topic is beginning to be examined in elasmobranchs, and it is unknown whether new information is added to and integrated with existing information or if switching from reliance on one sensory system to another occurs (Gardiner *et al.*, 2012). When one type of signal becomes obscured, e.g. vision at close range, others, e.g. electroreception, are likely to become increasingly important in directing the final stages of prey capture. Large blind spots in the visual field anterior to sharks are hypothesized to be almost completely overlapped by the sensitivity range of the electrosensory system, resulting in near-seamless sensory function (McComb *et al.*, 2009). Sharks have been observed to use at least two signal types in tandem, e.g. odour and water flow (Gardiner and Atema, 2007), or to have one type override another. For example, blue sharks, *Prionace glauca*, and dusky smoothhound, *Mustelus canis*, preferentially bit at a prey-simulating dipole rather than the prey odour source (Kalmijn, 1971).

Recent work indicates that certain sensory modalities direct specific prey-capture behaviours. Bonnethead sharks, *Sphyrna tiburo*, could locate but not accurately line up to capture food without vision, and could not efficiently strike to ingest food in the absence of electrical stimuli (Gardiner, 2011). The nurse shark, *Ginglymostoma cirratum*, required olfactory signals for food ingestion to occur even though orientation to the prey was successful with other stimuli (Gardiner, 2011). In the blacktip shark, *Carcharhinus limbatus*, both visual and olfactory cues were necessary for efficient prey location and capture (Gardiner, 2011). More generally, differences in sensory system morphology and development of the associated regions of the brain suggest a trade-off between vision and electroreception, where oceanic pelagic species may rely more heavily on vision and coastal benthic-feeding species on electroreception (Kajiura *et al.*, 2010). Variation exists, however, and other sensory cues may play an integral role for some species, such as odour in the nurse shark. Use of multiple sensory modalities in locating and capturing prey suggests that the most effective bycatch reduction approach, for at least some species, may involve combinations of strategies simultaneously directed at more than one sensory system.

## Signals produced by fishing gear

Whether it involves bait or not, every type of fishing gear emits a variety of signals when deployed underwater. At a minimum, these signals will include hydrodynamic disturbances and sounds created by the gear and/or the vessel deploying gear, and could also include odours, visual signals, and electrical cues. Any gear in contact with the substrate (e.g. weighted gill nets, bottom trawls, and demersal longlines), may generate various sounds, vibrations, and increased turbidity. Bait will contribute additional chemical and visual cues. Any entangled, hooked, or fouling organism may emit a variety of signals, including odours, hydrodynamic disturbances, struggling sounds, visual signals, and bioelectric fields. Here, we outline potential sensory signals emitted by various types of fishing gear that elasmobranchs may encounter and introduce potential bycatch reduction modifications to each gear type as topics for future research (summarized in Table 1).

**Table 1.** Potential applications of new and existing bycatch reduction technology by fishing gear and elasmobranch sensory modality

Sensory modality	Baited hook and line (longline)	Gill net	Trawl	Purse seine
Olfaction	Surfactants, semiochemicals	Surfactants, semiochemicals		Remote attraction/bait stations
	Bait type			
	Dead sharks			
Hearing	Not recommended			
Vision	Light sticks: wavelength and flicker	Net illumination	Flashing lights	
	Bait colour	Net colour		
	Leader type/colour	Predator models		
	Dead sharks			
Mechanosensory lateral line/pit organs			Water jets	
Electrosensory	Magnets, lanthanide metals, battery-powered electric devices	Powered electric field ‘barrier’	Electric pulse generators	
		Magnetic field ‘barrier’		
Other		Pre-net fence (tactile)		

Some of these may potentially be applied to other gear types, and all require additional research and development.

### Net fishing gear

**Figure 2.**
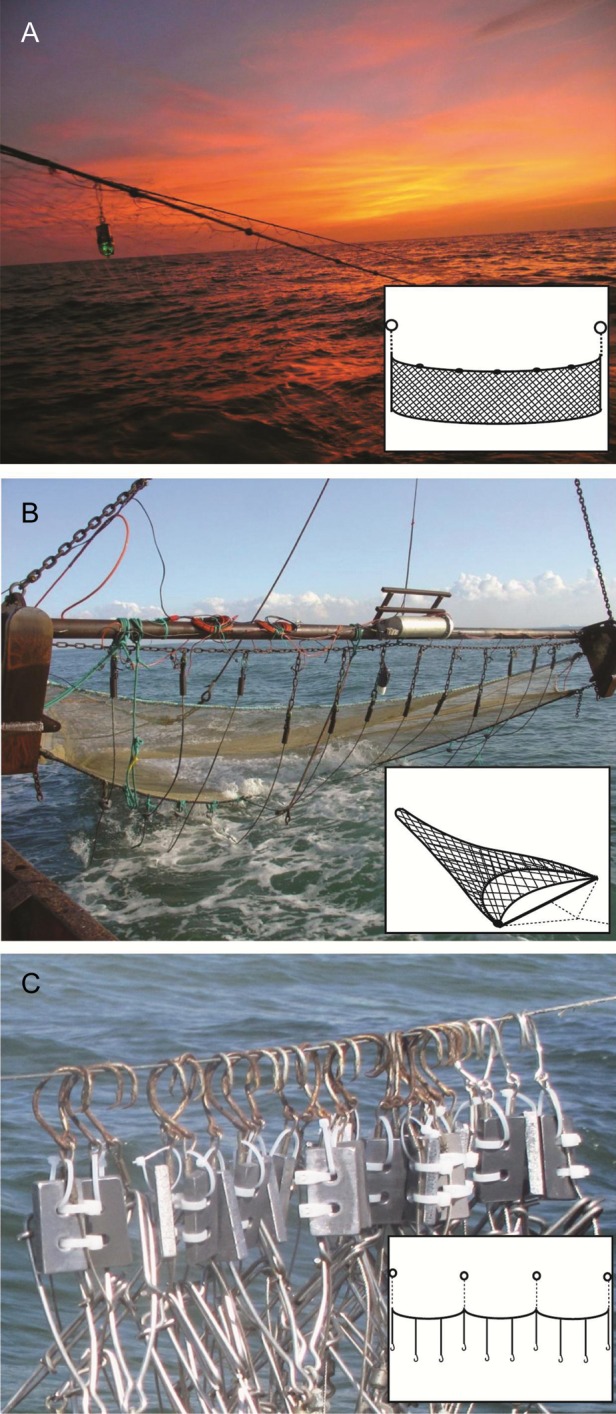
Examples of sensory-based deterrents attached to fishing gear. (**A**) Illuminated gill net (photograph credit Jesse Senko). (**B**) Beam trawl fitted with electric pulse generator, electrodes, and raised ground rope (Hovercran shrimp pulse trawl, photograph credit ILVO, Belgium). (**C**) Longline gear with lanthanide metal secured near hook (photograph credit Kieran Smith). Inset illustrations show examples of each fishing gear type when deployed

Gill nets are typically designed to be difficult to detect visually, although some are outfitted with various colours to attempt to select for certain target species or deter others (e.g. Melvin *et al.*, 1999). Increasingly, gill nets are equipped with high-frequency acoustic pingers, and other modifications, including illumination and stiffer, acoustically reflective materials, continue to be evaluated (Northridge *et al.*, 2003; Werner *et al.*, 2006; Larsen *et al.*, 2007; Trippel *et al.*, 2009; Bordino *et al.*, 2013). Stiffer materials or increased tension can decrease the risk of entanglement of elasmobranchs and other species (e.g. Thorpe *et al.*, 2001). Signals providing early warning of the presence of the net, e.g. visual or electromagnetic cues, may reduce elasmobranch interactions with gear and could override attractive signals produced by captured organisms. Visual enhancements, including lights affixed to nets (Fig. 2A), nets constructed of photoluminescent materials, or predator models, warrant further testing for their influence on elasmobranch and target species catch (see Wang *et al.*, 2010). The effects of different colours of nets on elasmobranchs in artisanal and commercial fisheries are largely unknown. Electrical barriers affixed to the net, either powered or magnetic, could repel elasmobranchs, preventing entanglement. The development of a pre-net ‘fence’, consisting of vertical float lines attached to a weighted line in front of the gill net, may provide visual and tactile cues to keep larger animals from becoming entangled, while smaller target species pass between float lines into the net (M. Kobza, personal communication).

Purse seine gear is commonly set over FADs (or organisms such as whale sharks) that can provide hydrodynamic, sound, and odour signals from aggregating fishes and fouling organisms. In one study, odour was suspected to be the major cue for fish to locate FADs, but no difference was found for fish returning to a FAD from downstream or cross-stream starting points, leading the researchers to suggest sound as potentially important (Dempster and Kingsford, 2003). Once the purse seine begins to be deployed around or near the FAD, additional hydrodynamic, chemical, and sound signals are likely to be present. The use of multiple FADs to facilitate species segregation (Schaefer and Fuller, 2011) and/or displaced attractants (e.g. bait stations; Dagorn, 2010) may help to reduce the number of elasmobranchs captured by a purse seine net. Within the net itself, both target and non-target species have been observed to segregate by size class and species, indicating potential for selective release of non-target species and size classes (Muir *et al.*, 2012). The use of sensory cues directing bycatch organisms toward escape routes both before and after setting the net show promise and warrant further research (Dagorn *et al.*, 2012).

Trawl gear dragged along the bottom causes sound, vibration, and hydrodynamic disturbance, and could be detected visibly, depending on light levels and turbidity, which may be increased by the gear itself. Entrained organisms may emit additional chemical and hydrodynamic signals. Trawl gear not in contact with the bottom would produce similar signals without increases in sound, vibration, and turbidity from bottom stirring. For a resting elasmobranch being approached by mobile gear, such as a trawl, signals prior to contact with the gear are likely to be limited to sound and vibrations, followed by visual signals of suspended sediments or the gear itself, because the animal may be upstream of chemical trails. Queirolo *et al.* (2012) observed sharks and skates to be fairly passive around the mouth of the trawl, possibly relying on camouflage to ‘hide’, staying motionless or swimming no faster than the trawl itself upon contact. Inter-specific differences in shark behaviour around approaching gear have been observed (Lorance and Trenkel, 2006), and herding, which is visually mediated in flatfish (Ryer *et al.*, 2010), can also occur in skates (Winger *et al.*, 2010). Signals including visual (lights), hydrodynamic (water jets), or electric fields could provide an early warning system to benthic elasmobranchs, which may increase reaction time to enable avoidance of approaching gear. Polet *et al.* (2005) demonstrated that electric pulse generators positioned at the mouth of a trawl (Fig. 2B) successfully caused brown shrimp, the targeted species, to jump upwards, allowing the ground rope to be raised by 10–15 cm with no reduction in shrimp catch. Raising the ground rope allows fishes and other invertebrates to escape under the net and results in less benthic damage (Polet *et al.*, 2005). Although not encountered in the above study, the electric pulse itself may alert elasmobranchs to the approaching gear and facilitate escape either below or away from the trawl.

### Baited fishing gear

Bait exudes chemical and visual signals, although the lines themselves can be difficult to detect visually. During deployment of longlines, activity from seabirds may cause additional sounds and disturbances. When baited gear, such as longlines, is deployed, we suspect that elasmobranchs are more likely to approach from downstream, where chemical signals are available. Pelagic sharks rely heavily on vision to locate prey, thus several visual signals warrant further experimentation for use on pelagic longlines, including light stick colour and flicker frequency, bait dying, and leader type or colour. Anecdotal evidence also suggests that the presence of dead sharks on or near fishing gear can decrease subsequent shark catch (S.J.B. Gulak, personal communication). The extent of chemical vs. visual signals contributing to this observed avoidance response is unclear, and controlled experiments are necessary. Several sharks feed on other elasmobranchs, suggesting that chemicals produced by freshly dead individuals may, in some cases, be attractive rather than repulsive, thus further understanding of chemicals produced by freshly dead vs. decaying sharks and the effects of these on nearby elasmobranchs is needed. Research into developing electric/magnetic deterrents for baited gear appears most promising for coastal demersal longlines, where bycatch species are more likely to rely heavily on electroreception (Fig. 2C).

The likelihood of striking at bait depends on a variety of environmental and biological variables, including both feeding motivation and the ability to locate the bait successfully (Stoner, 2004). The likelihood of becoming hooked also depends on feeding behaviour, including how the bait and hook are ingested, which may be influenced by hook type, placement within the bait, and bait type. Detailed knowledge of elasmobranch interactions with bait and fishing gear, however, are lacking.

## Feeding behaviour and ecology

While it is not within the scope of this review to present a comprehensive overview of elasmobranch feeding ecology and behaviour, we provide a brief background in the context of potential interactions with fishing gear.

### Overview of prey-capture mechanics/strategies

Elasmobranchs exhibit a variety of feeding and prey-capture strategies that vary with habitat and prey type. Strategies include filtering, ambushing, disabling, digging up, and chasing down prey, while capture may include suction and/or ram feeding, with various degrees of jaw protrusion (Motta and Huber, 2012). Prey capture, from mouth opening (expansion) to closing (compression), is typically rapid (100–400 ms), with variation associated with species and prey type (Motta *et al.*, 2002). The feeding apparatus of sharks is relatively simple in comparison to teleosts, and consists of 10 cartilaginous elements that make up and support the jaw (Motta, 2004). Regardless of whether suction or ram feeding occurs, bait and hooks can either enter the buccal cavity or become lodged in the jaw. Sharks are significantly less likely to survive when the hook is swallowed vs. embedded in the jaw (Campana *et al.*, 2009). Both hook size and hook shape can influence the likelihood of swallowing in teleosts and elasmobranchs (Bacheler and Buckel, 2004; Campana *et al.*, 2009), and hook material can affect how long the hook remains in the jaw if the line is cut. Circle hooks were developed to reduce catch rate and swallowing of hooks by sea turtles. While the effects of circle hooks on shark catch rates have been mixed, their use generally reduces at-vessel mortality of both sea turtles and elasmobranchs when compared with J hooks (e.g. Watson *et al.*, 2005; Afonso *et al.*, 2011; Curran and Bigelow, 2011, Godin *et al.*, 2012). Whether the elasmobranch swallows the bait whole or takes multiple bites may be a function of mouth size, bait type, or additional inter-specific differences. The differences in mouth shape, size, and bait ingestion mechanics between elasmobranchs and target species, as a means to reduce bycatch, have not yet been investigated.

### Behavioural ecology of elasmobranchs during feeding events

Sharks are generally considered intermittent, asynchronous, opportunistic feeders; however, many species are highly specialized for limited prey types, and most batoids and filter feeders feed more continuously (Motta and Wilga, 2001; Wetherbee and Cortés, 2004; Motta and Huber, 2012). Most elasmobranch species show evidence of being opportunistically selective feeders, choosing preferred prey when it is available (Wetherbee *et al.*, 1990). Many species feed in a solitary manner; however, some form large aggregations and employ co-ordinated efforts to herd prey (See Motta, 2004). Group feeding can provide advantages in terms of locating prey more efficiently, but can also introduce social and competitive factors, which may influence foraging success and prey selectivity. Aggressive feeding aggregations can involve groups of six to hundreds of sharks and may include indiscriminate feeding, accelerated speed, and injury to individuals (e.g. Nelson, 1969). Both the presence of potential competitors and increased hunger level have been suggested as factors that decrease the success of shark deterrents in field and laboratory settings, indicating the need for different strategies for solitary- vs. group-feeding species (Tallack and Mandelman, 2009; Jordan *et al.*, 2011; Robbins *et al.*, 2011).

### Behavioural ecology of non-feeding elasmobranch aggregations

Elasmobranch species have been observed to form aggregations for several reasons other than feeding. Schooling or shoaling behaviour can be associated with reproduction or refuging (Klimley and Nelson, 1984). Dominance hierarchies in shoals of sharks appear to be established by size and/or sex, and many species form separate aggregations based on size and/or sex (see Bres, 1993). Many of these aggregations are associated with environmental structures, such as reefs, sea mounts, or FADs, and disperse at regular intervals, such as night-time foraging (Bres, 1993; Filmalter *et al.*, 2011). Species segregation may play a key role in future bycatch reduction strategies for purse seines (Muir *et al.*, 2012). Aggregations of elasmobranchs (and other species) are highly vulnerable to fishing activities, particularly when large nets capable of entrapping entire shoals are used, and high catch numbers can lead to inflated population estimates (Fordham *et al.*, 2006).

## Species examples

Sensory system anatomy, feeding ecology, and prey-capture strategies vary widely among elasmobranchs, suggesting the need for more species-specific bycatch mitigation strategies. Here, we discuss in more detail a few species of US and/or international concern, for which regional or global attention to bycatch reduction is warranted. Species were chosen based on one or more of the following factors: (i) conservation concern; (ii) potential for successful bycatch reduction through the use of deterrents and/or gear modifications aimed at reducing interaction with fishing gear; and (iii) high or unknown at-vessel and/or post-release mortality.

### Scalloped hammerhead

Populations of hammerhead shark species are estimated to have declined by up to 85% since the 1980s in some regions, such as the northwest Atlantic ocean (Hayes *et al.*, 2009; Jiao *et al.*, 2009). The scalloped hammerhead (*Sphyrna lewini*) is currently listed by the IUCN as endangered globally and is largely taken as bycatch by pelagic longline and gill-net fishing vessels, although they are also captured by trawls, purse seines, bottom longlines, and inshore artisanal fisheries, particularly at smaller size classes (Baum *et al.*, 2007; Amandè *et al.*, 2010). High at-vessel mortality has been documented for scalloped hammerheads in pelagic longline fisheries (61%; Beerkircher *et al.*, 2002), demersal longlines (91.4%; Morgan and Burgess, 2007), and gill nets (93%; J. Carlson, unpublished data).

As a result of their unique head shape and corresponding sensory arrangements, hammerheads have been the subject of considerable sensory-related research. The expanded cephalofoil has been suggested to provide sensory and manoeuvrability advantages, in addition to its occasional use as a restraint for capturing benthic prey (Strong *et al.*, 1990; Kajiura *et al.*, 2003, 2005; McComb *et al.*, 2009). The location of eyes and nares farther from the mid-line of the body may improve binocular vision and tracking of odour plumes, respectively (Kajiura *et al.*, 2005; McComb *et al.*, 2009). Head shape does not appear to provide hammerhead sharks with enhanced electrosensitivity, although it does increase their lateral search area, which may be particularly useful when scanning for benthic prey (Kajiura and Holland, 2002). Scalloped hammerheads have been observed to disperse from aggregations near a sea mount to feed at night (Klimley, 1988), suggesting that bioluminescence and/or senses other than vision are important for tracking prey.

Based on their sensory systems and ecological roles, we suggest research into multiple sensory modalities to reduce bycatch of scalloped hammerheads and other species that share similar sensory characteristics. For example, we recommend studies investigating visual (e.g. light stick) and chemical cues, as well as electric/magnetic deterrents, which have already demonstrated some success. Scalloped hammerheads avoided a magnetic field created by ferrite magnet blocks (Rigg *et al.*, 2009), and lanthanide metal alloys significantly reduced catch of this species on longlines (Hutchinson *et al.*, 2012). Another species of concern, for which a similar bycatch reduction approach may be successful, is the dusky shark, *Carcharhinus obscurus*, which also has a high number of electrosensory pores (Kajiura *et al.*, 2010) and has demonstrated avoidance of electrical barriers (Smith ED, 1991). In contrast, the more pelagic blue shark, *Prionace glauca*, has a low number of electrosensory pores and probably relies more heavily on vision and olfaction to locate prey (Kajiura *et al.*, 2010). Lanthanide metal deterrents have failed to reduce blue shark catch rates, but visual and chemical bycatch reduction strategies may have more success (Hutchinson *et al.*, 2012).

### Smalltooth sawfish

Listed by the IUCN as critically endangered, the sawfishes are one of the most threatened families of fishes, having experienced dramatic population declines and near extirpation from areas where they were once common (Adams *et al.*, 2006; Compagno *et al.*, 2006; Charvet-Almeida *et al.*, 2007). For example, the population of smalltooth sawfish, *Pristis pectinata*, in US waters is essentially restricted to southern Florida (Simpfendorfer *et al.*, 2008). Fishing activities are the greatest threat to this species (Adams *et al.*, 2006). Smalltooth sawfish are taken as bycatch both in bottom longline fisheries and in shrimp trawls, where high mortality is estimated (National Marine Fisheries Service, 2012).

Owing to the high tendency of the saw-like rostrum to become entangled, preventing contact with fishing gear and ensuring safe release if captured are major objectives in sawfish recovery efforts. Sawfish have extensive electrosensory and lateral line systems extending out along the rostrum, which is used both to sense and to manipulate prey (Wueringer *et al.*, 2011a,b, 2012). Observations of sawfish interacting with trawl gear, such as those obtained by video, are crucial for devising effective ways to avoid sawfish capture and minimize mortality. If sawfish remain relatively passive until contact occurs with trawl gear, as observed in other elasmobranchs (Queirolo *et al.*, 2012), an early warning system including water jets and/or electric pulses may facilitate timely reaction and gear avoidance. The combined effects of electric pulses and a raised ground rope could significantly reduce bycatch of many species in addition to sawfish. If capture occurs, catch of narrow sawfish, *Anoxypristis cuspidate*, in Australia has been shown to decline with use of TEDs in trawl nets (Brewer *et al.*, 2006), although post-release condition is unknown. Although TEDs are also used in Florida waters where smalltooth sawfish occur, the effects of these on sawfish catch and mortality are not known (J. Carlson, personal observation).

### Blacknose shark

The blacknose shark, *Carcharhinus acronotus*, is currently listed by the IUCN as near threatened globally, has experienced a 25% population reduction between the 1950s and 2006 (Siegfried and Brooks, 2007), and is considered over-fished, with over-fishing occurring in the Atlantic ocean (National Marine Fisheries Service, 2011). This species is targeted by some fisheries (Carlson and Bethea, 2007) and is also caught as bycatch in teleost gill net and shrimp trawl fisheries, particularly as juveniles (Shepherd and Myers, 2005; Siegfried and Brooks, 2007).

Although little is known about blacknose shark behaviour, visual characteristics suggest that this species is more active during low-light, crepuscular, or nocturnal periods, and it has a 360 degree dorsal and ventral visual field, with frontal binocular vision (McComb *et al.*, 2009, 2010). Like other coastal pelagic Carcharhinid species, blacknose sharks have a relatively high number of electrosensory pores, suggesting the importance of electroreception in the perception of their environment and location of prey (Kajiura *et al.*, 2010). Capture of blacknose sharks in gill nets is highly dependent on mesh size (Carlson and Cortés, 2003), and modifying this, along with sensory-based approaches, may reduce bycatch in gill-net fisheries. Known visual and electrosensory characteristics suggest that net lighting and electrical deterrents are particularly promising lines of research to pursue for bycatch reduction of blacknose sharks in gill nets. These modifications, as well as pre-net fences, discussed earlier, warrant further study to determine their effectiveness in reducing contact of non-target species with gill nets.

### Manta rays

The giant manta, *Manta birostris*, and reef manta, *Manta alfredi*, were only recently recognized as separate species (Marshall *et al.*, 2009), and are often confused with devil rays of the genus *Mobula*. Thus, historical fishery records mix or confuse these species, but both mantas are listed by the IUCN as vulnerable (Marshall *et al.*, 2011). Global populations of mantas are estimated to have decreased by at least 30%, with declines of up to 80% in some regions (Marshall *et al.*, 2011). These species are taken as bycatch in longline (Beerkircher *et al.*, 2002) and, more commonly, in net fisheries, such as purse seines (Coan *et al.*, 2000; Amandè *et al.*, 2010), pelagic trawls (Zeeberg *et al.*, 2006), and gill nets (Trent *et al.*, 1997; White *et al.*, 2006).

Manta rays filter feed on planktonic organisms, either near the surface or at depth, using unfurled cephalic lobes to direct food and water into the mouth. Although few quantitative data on manta sensory anatomy and detection capabilities exist, the electrosensory system appears markedly reduced, while the lateral line is extensive and highly branched (Chu and Wen, 1979). The eyes of mantas are relatively small and oriented for forward, lateral, and downward vision. A captive manta displayed feeding responses to visual and olfactory stimuli (Ari and Correia, 2008), while electromagnetic and olfactory cues are likely to be important in navigation during migrations. Owing to their large size, mantas may be visible to fishers and should be avoided as a first step toward reducing interactions with gear. Manta and mobulid rays were more frequently captured by European purse seiners in the Atlantic ocean in sets on free-swimming tuna rather than in sets on FADs (Amandè *et al.*, 2010). This trend is opposite to that observed for overall bycatch, and for sharks, where 91% were caught in sets with FADs (Amandè *et al.*, 2010).

Priorities for manta ray bycatch reduction include more detailed and quantitative knowledge of their sensory biology, along with observations of mantas interacting with fishing gear. We suggest the use of side-scan sonar, video, or observers to look for any patterns, such as upward or downward swimming, that might be useful in determining avoidance or release strategies for mantas once inside purse seine nets. Observations such as these have contributed to the development of highly effective dolphin bycatch reduction techniques (e.g. Barham *et al.*, 1977; Francis *et al.*, 1992), and are currently being developed for release of sharks from purse seines (Itano *et al.*, 2012). In addition, remote bait stations, such as those currently being explored for sharks (Dagorn, 2010), may assist in diverting mantas away from fishing areas; however, more information on how mantas locate prey and associate with tuna schools is needed.

## Conclusions, new directions, and challenges

Over-fishing, including excessive mortality from bycatch, is the largest threat to elasmobranch populations. According to the IUCN, of the 563 elasmobranch species not considered data deficient, 55% are categorized as threatened or near threatened (IUCN Redlist). Concern over depleted elasmobranch populations and waste associated with bycatch has led to a surge in bycatch reduction-related research. Despite attempts to understand and exploit elasmobranch sensory biology for prevention of shark attacks since 1945, no deterrent has, to our knowledge, proved effective for all species and situations tested (Gilbert, 1968; Sisneros and Nelson, 2001; O'Connell *et al.*, 2012). Elasmobranchs are a diverse group of over 1100 species, exhibiting wide ranges in habitat, behaviour, ecology, sensory biology, and neurobiology (Gardiner *et al.*, 2012; White and Last, 2012; Yopak, 2012). For these reasons, we suggest a species- and fishery-specific approach that builds upon current knowledge of sensory biology and behaviour of target and non-target species. While a universally effective deterrent may be unlikely, greater understanding of sensory capabilities and feeding behaviour may shed light on deterrents or combinations of deterrents that will be effective in reducing elasmobranch interactions with particular fisheries. This information, combined with more detailed knowledge of fishing practices (made available through observer programmes, as well as co-operation and collaboration with the fishing industry) can be integrated to develop more effective elasmbranch bycatch reduction strategies.

In order to expand the field of elasmobranch bycatch reduction research, we have identified several opportunities for future research and collaboration. Improving our understanding of how various elasmobranch species sense and interact with fishing gear and how this differs from target species is a key priority. Detailed knowledge of sensory biology and behaviour is lacking for many elasmobranch species, including several that make up large components of bycatch from various fisheries. Combinations of anatomical, behavioural, and field studies are ideal, but not feasible for some species (e.g. rare/protected species and/or those that do not survive in captivity). Identification of specific sensory cues broadcast by a type of fishing gear and evaluation of when, how, and which sensory information is used by a given species should lead toward effective solutions for reducing gear interactions. Several new and existing technologies should undergo further testing for use in elasmobranch bycatch mitigation (Table 1). Selection of which technology is likely to be most effective will depend on the fishery and species involved, particularly for fisheries with high bycatch rates, where one species dominates the bycatch, or when mortality associated with a fishery poses significant risks to threatened populations.

Behavioural observations of elasmobranchs prior to and during capture are needed in order to suggest promising techniques for minimizing vulnerability to fishing gear. Despite a vast body of research on teleosts (e.g. Somerton, 2004; Broadhurst *et al.*, 2007; He, 2009), few studies have directly or indirectly investigated elasmobranch behaviour around fishing gear. Strategies to increase gear selectivity and reduce bycatch of non-target teleost fishes often seek to evaluate and exploit species-specific behavioural tendencies (Breen *et al.*, 2004; Walsh *et al.*, 2004; Videler and He, 2010). For example, in tuna longlining, where shark bycatch occurs, analysis of differences in mouth shape, approach, and ingestion of bait may reveal new hook designs, in addition to J and circle hooks, or novel bait hooking strategies to decrease bycatch. Technology for video or sonar surveillance of trawling and other fishing operations is an important tool for understanding when and how various species, including elasmobranchs, react to approaching gear (e.g. Queirolo *et al.*, 2012). Use of this technology should accompany catch data as new bycatch reduction methods are tested for a more complete understanding of how gear modifications and deterrents influence the behaviour of both target and non-target species.

In addition to adopting an integrative approach, including modelling, laboratory, and field tests to understand consequences of gear alterations on both target and non-target species (Molina and Cooke, 2012), we emphasize the need for greater collaboration among researchers studying bycatch reduction of different taxonomic groups. When testing gear modifications in the field, recording detailed species-specific catch data is vital to the overall goal of bycatch mitigation. Many bycatch reduction studies focusing on other taxa list elasmobranchs among the catch, yet do not include detailed analyses of the effects of gear modifications on elasmobranch catch rate and/or mortality (with notable exceptions, such as Watson *et al.*, 2005). This missing information could provide valuable insights into types of modifications that influence elasmobranch vulnerability to fishing gear. Also, greater collaboration among scientists should streamline efforts to develop technologies that reduce bycatch of all non-target species, rather than decreasing the catch of one species or group while increasing the catch of another.

Bycatch reduction strategies aimed at each sensory system have inherent advantages and challenges based on the nature of the signal and how it can be produced. Many gear modifications and deterrents present logistical, economic, and environmental challenges. For example, chemical deterrents may disperse too rapidly or slowly to be effective, and certain chemicals may pose a pollution risk or could negatively affect marine organisms (see O'Connell *et al.*, 2012). In contrast, chemical deterrents may be fairly cost effective for mass production and be relatively simple to integrate into current fishing practices (e.g. pre-treated baits). Any of the proposed bycatch mitigation methods will be likely to meet with less resistance from the fishing community if their benefits (such as increased or unaffected target catch and/or decreased depredation and gear damage) outweigh their costs in terms of materials and time spent modifying gear.

In addition to development of new techniques to prevent elasmobranch bycatch, increased motivation and incentives for policymakers and the fishing industry to prioritize elasmobranch bycatch reduction are necessary. The demand for shark fins and other elasmobranch products is high and growing in some markets, resulting in disincentives to research, mandate, employ, and enforce the use of methods to minimize elasmobranch bycatch. Several elasmobranch bycatch reduction techniques (e.g. using fish instead of squid bait) have already proved useful, yet have not achieved widespread implementation (Gilman *et al.*, 2008). Economic and political pressures often outweigh scientific advice in fisheries management arenas, contributing to the general state of over-exploitation of global fisheries (e.g. Gilman, 2011). Despite challenges to international co-operation in the management of fisheries and bycatch, broad changes have been relatively successful (e.g. the moratorium on the use of high-seas drift nets and the implementation of TEDs in trawls), lending hope for future progress toward more sustainable fishing. As the human population and demand for seafood continue to rise, development of sustainable fisheries, with minimal bycatch, are increasingly important. Research that integrates the sensory biology and behavioural characteristics of elasmobranch species can help to improve gear selectivity, support more effective fisheries management, and facilitate recovery of threatened species.
